# *Through the Patients’ Eyes*: The Experience of End-Stage Renal Disease Patients Concerning the Provided Nursing Care

**DOI:** 10.3390/healthcare5030036

**Published:** 2017-07-21

**Authors:** Areti Stavropoulou, Maria G. Grammatikopoulou, Michail Rovithis, Konstantina Kyriakidi, Andriani Pylarinou, Anastasia G. Markaki

**Affiliations:** 1 Department of Nursing, Technological Educational Institute of Crete, Estavromenos, Irakleio 71004, Greece; aretistavropoulou@gmail.com (A.S.); rovithismihail@gmail.com (M.R.); 2 Department of Nutrition & Dietetics, Alexander Technological Educational Institute, Sindos 57400, Greece; maria@nutr.teithe.gr; 3 Department of Nutrition & Dietetics, Technological Educational Institute of Crete, Estavromenos, Irakleio 71004, Greece; kwnstantinakyr@hotmail.gr (K.K.); andrianapilarinou@hotmail.gr (A.P.)

**Keywords:** chronic kidney disease, hemodialysis patients, nursing care, qualitative study, patient experience, ESRD, renal disease, nephrology nurse, nephrology care

## Abstract

Chronic kidney disease is a condition that affects both the physical and mental abilities of patients. Nursing care is of pivotal importance, in particular when end-stage renal disease (ESRD) patients are concerned, since the quality of the provided care may severely influence the patient’s quality of life. This is why it is important to explore patient experiences concerning the rendered care. However, limited up-to-date studies have addressed this issue. The aim of the present study was to stress the experiences of ESRD patients concerning the provided nursing care in the hemodialysis unit at the University Hospital in Heraklion, Crete. A qualitative methodological approach was used, based on the principles of phenomenological epistemology. Semi-structured interviews were conducted, and open-ended questions were applied to record *how* patients experienced the rendered care during dialysis. The recorded data were analyzed via qualitative content analysis, which revealed three main themes: ‘Physical Care’, ‘Psychological Support’ and ‘Education’. Patients’ views were conceptualized into sub-themes within each main theme. The interviews revealed the varied and distinct views of ESRD patients, indicating that the rendered care should be individualized.

## 1. Introduction

Chronic kidney disease (CKD) is currently affecting 18.4% of the European population [1], with end-stage renal disease (ESRD) patients in particular receiving a disproportionate share of health care resources and spending a great amount of time using treatment facilities. Since the performance of the first hemodialysis in a human by Hass in 1924, it still remains the most preferred treatment for ESRD today [2,3]. According to the latest European Renal Association - European Dialysis and Transplantation Association registry data [3], in Greece, there have been an estimated 1.1 million CKD patients (10% of the total population), with 13,359 of them being ESRD patients. Among ESRD Greek patients, approximately 75% (10,029) are on hemodialysis [4], indicating that this specific renal replacement therapy (RRT) is the therapeutic option for the majority of the Greek ESRD patients. Consequently, the financial burden on the Greek health care system, along with ESRD patients’ burden, is extremely high.

The objectives of care for patients on hemodialysis include providing sufficient dialysis, maintaining vascular access, ensuring adequate nutrition, minimizing hospitalization, and prolonging life and its quality [5]. Nursing care, on the other hand, involves interactive actions built on the ethical dimension between the caregiver and the client [6]. Hemodialysis in particular requires specialized nursing care that extends beyond technical knowledge and the suggested Nursing Care Plans (NCP). It includes therapeutic and interpersonal relationships, as well as prompt responses to physical symptoms, functional limitations, psychosocial disruptions, and knowledge needs [6,7].

According to Kimmel [8], hemodialysis is a lifelong treatment that significantly and sometimes adversely affects patients’ physical and mental abilities, with depression, anxiety and fatigue being common issues [9,10]. There are also a plethora of additional stressors, including biochemical imbalance, physiological changes, neurological disturbances, cognitive impairment, and sexual dysfunction, all associated with ESRD [11]. For hemodialysis patients who spend a considerable portion of their life receiving treatment, nursing care is of pivotal importance, and the identification of the bottlenecks hampering nursing care would contribute to an ameliorated relationship between nephrology nurses and patients and inevitably, to a better quality of life for the latter. However, literature is scarce on the experience and beliefs of ESRD patients receiving care.

The nursing care offered on hemodialysis patients entails unique characteristics in relation to other nursing plans. Frequent patient visits (usually three times a week) lead to an increased proximity to the nursing staff [12]. Moreover, the clinical environment where hemodialysis nurses are called to serve is demanding and stressful, leading many nurses to develop burnout syndrome [13,14,15]. In particular, one of the main obstacles to the provision of nursing care among ESRD patients concerns the increased workload, augmented by various factors such as the need for different care services, the limited number of employed nurses and other health professionals, and the lack of a strategy for systematic education and guidance of patients [16]. However, according to a cohort study performed among 320 hemodialysis patients from 14 general hospitals situated in Athens and the peninsula of Peloponnese, the majority of patients reported satisfaction with regards to the availability of information prior to the initiation of hemodialysis and the provision of nursing care. Moreover, patients were very/extremely satisfied with the provided care in terms of the duration of the changing sera/intravenous solutions, the kindness and empathy expressed by nurses, as well as from their professionalism and applied techniques, throughout the provided care [17]. However, some international studies have reported a lack of information concerning the available treatments [18], and low satisfaction from the provided nursing care [19].

In Greece, the nursing care provided to ESRD patients is of particular importance due to the large proportion of patients suffering from chronic renal failure and the great number of those on hemodialysis. Additionally, the quality of the rendered care is associated with the role of nephrology nurses, with specialized expertise and patient–nurse interaction being important predictors affecting the quality of the provided care. This is why patients’ experiences could provide valuable feedback for the nurses in order to reassess, set goals and improve the quality and safety of the provided care. In this context, the present study aimed to explore the experiences of hemodialysis patients concerning the provided nursing care, using a qualitative methodological approach, based on phenomenological epistemology.

## 2. Materials and Methods

A qualitative methodological approach based on phenomenological epistemology was used, as this allows researchers to explore patients’ lived experiences in detail.

### 2.1. Sample and Recruitment

The study was conducted at the University Hospital of Heraklion, in Crete, Greece, during the year 2016. Participants were recruited from the hemodialysis unit via a non-random purposeful sampling strategy, as the aim was to identify and involve patients whose experience of care during hemodialysis might produce valuable information on the topic. Inclusion criteria were (1) being an adult, (2) being on hemodialysis for more than a year, and (3) being able to understand and articulate effortlessly in the Greek language. All nurse managers and doctors at the hemodialysis unit were informed of the study’s purpose and were asked to identify potential participants matching the inclusion criteria. Each potential participant was addressed individually and informed of the study’s nature prior to participation. In total, 10 patients aged between 34–68 years old agreed to participate and provided informed consent. Patient characteristics are presented in Table 1.

### 2.2. Data Collection

All interviews were conducted by an experienced researcher in a quiet area chosen by the participant, either within the hospital or in the patients’ residence. Each participant provided oral permission prior to the interviews’ digital recording. Semi-structured, face-to-face interviews were conducted, facilitated by open-ended guide questions, in order to encourage patients to share their views and gain an in-depth understanding of their experience concerning nursing care during hemodialysis. The interviews lasted approximately 15–25 min for each participant, and the recorded interviews were then transcribed verbatim.

### 2.3. Data Analysis

Α qualitative content analysis was used to analyze the data. Words or phrases of the participants’ statements consisted the units of analysis. Common concepts and themes that reflected the experiences of patients were identified from analyzing the participants’ reports. Common concepts were grouped into categories. Comparing and contrasting the emerged categories led to the development of three main themes. Emerging sub-themes within each main theme reflected patients’ experience of the provided nursing care.

### 2.4. Ethical Considerations

Prior to the commencement of the study, the research proposal, a consent form, a participant information sheet and risk assessment form were submitted to the Research Ethics Committee of the University General Hospital, situated in Heraklion, Crete. Ethical approvals were granted by the Scientific Committee, as well as the nurse managers and the medical directors of the hemodialysis unit before the recruitment and data collection. All data were handled according to the Helsinki Declaration and its latter amendments. Voluntary participation and anonymity were emphasized, and participants were assured of confidentiality concerning their responses. Confirmation was provided that all information collected through the interviews would be used solely for the stated research purposes. Participants were also informed of their right to withdraw from the study at any stage without facing consequences affecting the routine care they received. Informed consent was provided prior to the interviews and all recordings and written information remained anonymous. Code names were given to all participants for better presentation of the results.

## 3. Results

Analysis of the interviews capturing patients’ experience of the care rendered in the hemodialysis unit revealed three main themes: (1) ‘Physical Care’, (2) ‘Psychological Support’ and (3) ‘Education’ (Table 2). Patients’ views and perceptions were conceptualized into sub-themes within each main theme. Table 3 stresses verbatim quotes defining the patients’ views on nursing and ambulatory care during hemodialysis, with the use of pseudonyms throughout.

### 3.1. Physical Care

Issues related to the physical care seemed to be of paramount importance for ESRD patients. Preservation of decency, nurses’ cautiousness and skillfulness and adherence to safe practices were indicated by the participants as important factors. Additionally, the importance of preserving dignity and empathy throughout the nursing process was stressed, as indicated by Moira’s statement (Table 3).

Additionally, the majority of participants focused the abilities and technical skills demonstrated by the nurses during the provision of care. Natalia stressed the importance of demonstrating and applying excellent practice during catheterization, whereas cautiousness and skillfulness appeared to be equally important in ensuring patients’ comfort. It also seemed that the responders viewed nurses’ cautiousness and skillfulness as distinct issues from affectionate care, as indicated by Glenn’s statement. The participants also referred to the factors that might impede nurses from applying the best practice such as the heavy workload (Alex), whereas Marvin argued that cautiousness was actually a matter of professional attitude and knowledge.

Concerning the third sub-theme, “Reassuring safe practices”, the safety and quality of equipment were identified as key issues in providing safe and quality care. Another interesting finding was that cautiousness, capacity, safety procedures and the quality of the equipment used were considered more important according to Steve than having an empathic and effective relationship with the staff. On the other hand, Marvin compared his experience on care in two different units, hinting at the equally important issue of being compassionate.

Participants additionally referred to environmental issues as part of the adequate quality of the physical care and reassuring therapeutic process. Adam, for instance, highlighted the importance of a responsive and reassuring environment.

### 3.2. Psychological Support

Patients’ experiences also focused on issues of psychological support in terms of provision of empathic care, the development of effective relationships with the staff, and good communication patterns. The first sub-theme concerned “Showing empathy and acceptance”, which produced either evolving feelings, as seen in Natalia’s statement, or more moderate experiences, as stressed by Steve and Elliot.

Concerning the interpersonal relationships with the nursing staff, participants associated the issue with concepts of acceptance, strength, support and mutual affection, as indicated by Natalia’s and Daniel’s statements. According to the first, the building of empathic relationships with other hemodialysis patients was additionally important, whereas Belinda pointed out several obstacles impeding the development of good relationships, such as the demanding hospital environment.

Issues regarding communication patterns seemed to be of major concern for the responders. Mostly, they expressed skepticism about communication practices, and some of them referred to issues that hindered effective communication between staff and patients. While Moira stressed the value of good communication, other responders focused mainly on the obstacles impeding effective communication from the staff. Although Alex believed that good communication consisted of a nursing duty, he experienced a different reality.

### 3.3. Education

Patients’ education appeared to be another issue of discussion and skepticism. Overall, responders felt that although education consisted of an important part of care, it was neglected and left upon each patient’s personal effort, as pointed out by Adam, Steve and Marvin.

Additionally, participants stressed the value of training provided by the staff. Adam and Belinda reflected the importance of receiving accurate and timely information by skilled and experienced nurses. Marvin, on the other hand, revealed the need to receive information on time. Moira’s experience focused on the absence of formal educational activities, and the effect this might have on patient anxiety and uncertainty for the future. Adam delineated the factors impeding the provision of formal patient education.

## 4. Discussion

The research herein identified three main themes of concern regarding nursing care among hemodialysis patients: physical care, psychological support and patient education. Multiple factors were associated with each domain, which contributed to understanding the provided nursing care from the patient’s point of view.

The rendered care involved personal dignity, nurses’ technical skills and safe practices, and the maintenance of a responsive environment. Hemodialysis patients in particular require constant physical and emotional support. Nurses, on the other hand, ought to understand patients, build trust and respond to their needs through encouragement [20,21]. To date, a number of studies have examined the experiences of CKD patients regarding the nursing care provided to them. In Kenya, ESRD patients reported neutral satisfaction with the provided care, mainly due to the way nurses attended to patients [22]. According to Chochinov and associates [23], ESRD patients face unique challenges as they move towards the end of life; simultaneously, the prevalence of depression is also high in patients with CΚD [24]. Additionally, patients are unwilling to become a burden or liability, and question their identity, pride, and self-image [25]. Thus, nurses should adopt an individualized approach to supporting psychological well-being and helping patients adapt to a changing lifestyle [21].

Nursing competencies and the assurance of safe practices were other important issues highlighted by the participants. A recent study in Iran revealed that ESRD patients expected nurses to attain the knowledge and skills to perform all care-related activities at ease [26]. Before meeting the patients’ needs, nurses require a certain understanding of technology, technical skills, knowledge, and experience [27]. The participants herein compared competencies, care protocols and skills between hospitals and nurses. In Kenya, ESRD patients noted the importance of equipment and the nurse–patient ratio as important factors affecting patient satisfaction [22]. Janssen and associates [28] explored the preferences of 4,518 patients undergoing hemodialysis. The findings showed that patients considered safety and safe practices as a more important element of their treatment than life expectancy [28]. According to a recent study on Greek ESRD patients, nurses’ technical and communication skills, competencies, timely intervention, and attitudes such as kindness, empathy, and respect for patients’ needs, diversities and professionalism, were essential features of the provided care [15]. However, it should be noted that the lack of human resources, guidelines, and regulations in certain hospitals often confuse nurses working in hemodialysis [27]. According to Garbin and Chmielewski [29], in order to provide safe and quality of care, nephrology nurses should fully understand the role of the health care team members and be able to allow other people to take care of their patients.

Research has showed that CKD patients experience emotional distress even in the earlier stages of disease progression [30] and in the majority, experience low feelings of personal control and low levels of illness coherence [31]. Patients have described the experience of hemodialysis as a daily struggle to accept illness and adjust to the treatment [32]. As Jonasson and Gustafsson [33] have noted, the challenge for nurses is to be able to support patients who are in a transitional phase of their life and be able to help patients regain lost stability and well-being.

The need to support patients psychologically has been highlighted in recent publications. Both the National Service Framework (NSF) for renal services [34] and the NSF for long-term conditions [35] indicate that care planning must recognize patients’ psychological needs in order to minimize mental health deterioration. According to Sedgewick [36], as well as Wright and Weinman [37], clinical psychologists can be a great asset in a multidisciplinary renal unit. However, it is important for nurses, who interact with patients more frequently, to obtain patients’ views on whether and when psychological support would be beneficial, as well as who they feel ought to provide it [38].

Leatherland [38] argued that an important action that all nurses should perform in order to ameliorate patient psychological health is to be available for patients to talk to. According to Swanson’s theory of caring [39], being with patients involves being emotionally present and facilitating their passage through life transformation by providing information, explanations, support, acceptance, emotion, alternatives, and feedback [39]. According to a qualitative descriptive phenomenological study conducted among 14 nurses at two distinct hemodialysis centers in Korea, nurses are not psychologically and emotionally inactive while providing care. On the contrary, they experienced sadness regarding patients’ lives, which involved enduring hemodialysis treatment, and they endeavored to establish good relationships and become comfortable with clients, their family and friends [40]. In the study herein, the participants greatly valued the supportive mechanism formed by the nursing staff and the other patients. As Auer [25] noted, for some patients, the regular trips to the hospital for treatment can be a welcome social activity.

Clinical practice guidelines for the treatment of kidney disease advocate the provision of education to patients [41,42]. Research has showed that timely and adequate patient education can balance modality selection [43,44], prepare for a better initiation of dialysis [45], and promote independence, while also encouraging self-management [46,47]. Recent advances in CKD patient education recommend the development of training programs based on the principles of the adult learning theory, which initially assesses individuals’ ability and willingness to learn [48,49]. Demonstration and instruction are the most widely used teaching activities. However, if different trainers are involved in the training process and each of them demonstrates things differently, this results in the patients becoming confused. In addition, instructions and demonstrations should be simple and concise [49].

According to a multinational study conducted by the European Kidney Patients’ Federation (CEAPIR) [37], CKD patients reported to be overall satisfied with the education received on their disease and treatment, although information seemed mostly to have been focused on one modality, with patients involved in modality selection being more satisfied with their treatment. However, studies on the USA using ESRD patients only have showed different results. According to Fadem et al. [16], ESRD patients are not uniformly advised about all possible treatment methods, which results in a fairly moderate satisfaction with their pre-treatment education. In the present study, patients identified the importance of personal effort on attaining adequate education and disease knowledge, exchanging information between more experienced patients and less experienced ones, and the nursing staff attaining disease-specific education. Harrison [50] argued that as far as patient education is concerned, many nurses lack the required skills, knowledge, and self-esteem; this is why tutoring nurses is essential in order to develop their ability and confidence [38]. In Kenya, ESRD patients valued greatly the post-dialyses guidelines received by nurses, as well as the assignment of a renal counselor to each patient and the availability of a nutritionist in the renal unit [22].

According to Kazley and associates [51], the majority of clinicians agree that there is typically a wide range of reactions among CKD patients. Additionally, because of various complications, as well as physical and psychological problems, ESRD patients are in need of continuous and comprehensive patient care to ensure adherence to therapy [26]. Our study presented the varied and distinct views of ESRD patients concerning nursing care during hemodialysis, and indicated that each patient should be treated accordingly and individually.

Qualitative research would provide valuable insight into the effectiveness of CKD patients’ views and perceptions from the provided nursing care. The cultural characteristics of the patients who participated in the study, as well as their strong dependence on the medical and nursing staff, which is common in the Greek health system, may have affected the variety of data, as well as prevented the participants from expressing themselves freely and sharing their concerns about the delivery and quality of the nursing care. Future studies could investigate whether specific components of the nursing care could be improved through targeted educational interventions in order to ameliorate nursing care quality.

## 5. Conclusions and Implications of Nursing Practice

The current qualitative analysis of the experiences of CKD patients could potentially facilitate our understanding of how specific characteristics and competences of nephrology nurses have a direct impact on the quality of the provided health care. This is imperative, as the common concepts and themes that reflected the experiences of patients revealed from this study, are often related directly to the performance and quality of the nursing care. This highlights the importance of patients’ views and perceptions as key performance determinants of healthcare delivery. The findings could help nursing students and nurses who lack experience of CKD care to understand patient needs and deliver care of better quality.

## Figures and Tables

**Table 1:** Participants’ demographic data (*n* or mean ± SD).

Men/Women (*n*)	7/3
Age (years)	56 ± 4
Marital status (married/single) (*n*)	6/4
Education (primary/secondary/tertiary) (*n*)	4/3/3
Employment (retired/self-employed/employee/unemployed)	5/2/2/1
Duration of hemodialysis (years)	5 ± 4

**Table 2:** Interviews’ main themes and sub-themes.

Physical Care	Psychological Support	Education
Preserving dignity	Showing empathy and acceptance	Personal effort
Demonstrating technical skills	Developing interpersonal relationships	Professional skill
Reassuring safe practices	Communicating effectively	
Maintaining a responsive environment		

**Table 3:** Verbatim quotes defining the patients’ views on nursing care during hemodialysis.

Theme	Sub-Theme	Quote	Patient
**1. Physical care **	1a. Preserving dignity	*It is annoying when someone is trying to make fun of you… I do not allow that to anyone… sometimes people they use black humor… it depends on their character and culture, but it’s annoying… I do not accept it… I want to preserve my dignity… I don’t want anybody to irritate me, or stress me, I want to be respected.*	Moira
	1b. Demonstrating technical skills	*It is important… the way that (a nurse) inserts the needle in your skin, the way she takes it out… it is important to have the skills to prevent vein damaging.*	Natalia
		*It happened twice… they (nurses) are either reckless or they do not know how to do it… I suffered during catheterization.*	Moira
		*… the way a nurse inserts the catheter in your vain… Sometimes the nurse is in a rush and damages my veins...*	Steve
		*Overall, nurses love us, but some of them are not skillful in using the needles, they try… but do not have the skills, damaging our arms. They (nurses) are not cautious when inserting the needle… I don’t know what they are thinking. They are present but at the same time they are absent… Most of the nurses are very experienced… They look at your arm, searching with their fingers for the best place to insert the needle and then they do it….*	Glenn
		*Some of us (the patients) complain about their (nurses) abilities and skills concerning catheterization… I understand the situation… It’s difficult for them… They (nurses) aim for the best, despite their workload, they are struggling every day in order to provide the best care.*	Alex
		*Some focus on simply doing their job, whereas others are completely indifferent. I have noticed that nurses want the easy way… so they put the needle at the same position. But this is wrong… the vain loses its elasticity. Normally they should insert the needle in different positions, but I do not know… it may be easier for them to use the same position… Some of them do not care and this is quite obvious... others are not interested in anything, and others just lack knowledge.*	Marvin
	1c. Reassuring safe practices	*The quality of the equipment is important. In some hospitals the equipment is better than others. Here, they have a blood pressure system that keeps checking every half hour… that is important.*	Moira
		*In another hospital where I was treated, the equipment was superior, however the environment was impersonal. What matters the most is following a right and safe procedure, ensuring that everything is sterilized and, if something wrong happens, to be looked after according to safety procedures… I prefer the nurses to be impersonal rather than to incapable… The incapable can cause you damage and I want to be safe… An impersonal nurse might be much better than one showing love but does not know how to provide care resulting in patients feeling unsafe…*	Steve
		*The quality of care here is high… People are friendlier and more compassionate. They love you more than the Italians… the Italians are more impersonal, but they are more consistent to following protocols.*	Marvin
	1d. Maintaining a responsive environment	*Around me there are several inpatients in a dire state… some of them close to dying. This affects me psychologically.*	Adam
**2. Psychological Support**	2a. Showing empathy and acceptance	*They (nurses) gave me so much strength… I returned to my house and I was thinking about seeing again those beautiful, lovely people… They care so much. They are responsible professionals and I know they care and know everything. They look after me… they do everything to make me feel comfortable.*	Natalia
		*Some care for you and others are sticking to a more professional attitude, they do not care what you have been through…*	Steve
		*What they do for me is satisfactory. I do not believe they can do anything extra. They do their work.*	Elliot
	2b. Developing interpersonal relationships	*I have no complaints…nurses and doctors, they love me and I love them back…*	Daniel
		*They accept me with so much love… people (nurses and doctors) that I have not met before. I did not want to start hemodialysis, instead, I wanted to die. But when they embraced me with such love, all these people… I will not forget their faces, their kindness. Awesome experience! They supported me psychologically. I see their faces and I think… they are all my children. Through all these I loved everyone in the unit, the patients too… I looked at them with so much empathy…*	Natalia
		*They are all very good to me and I admire their courage to struggle with so difficult situations*	Belinda
	2c. Communicating effectively	*Good communication… it is not perfect, but also not that bad. Everything is OK, when I need something, I have it. In general, it is not like being next to you all the time. I have lived this experience too, not here though… Some people are more emotional, they stand by you, helping a lot by discussing things… this helped me a lot psychologically when I was in a terrible state.*	Moira
		*It is their duty to communicate with the patients… we understand however the burdens. This is the reality…*	
		*The heavy workload impedes communication… doctors cannot be with you every single day. Sometimes you can realize the lack of communication… they (doctors) cannot stay next to you to ask how you are doing … They have a heavy workload, some are tired, others are very interested in you, others are a bit indifferent.*	Moira
**3. Education**	3a. Personal Effort	*They told us a few things, but they do not teach us every day… They gave us some advice and we are responsible for ourselves. There is an exchange of information between patients, the more experienced ones pass information to the newcomers… that’s how the others did it, so I do it the same way…*	Adam
		*I was asking things... Yes, when I went to the unit I had information, not enough, thus I was asking for more... I think that education is a personal effort... I trust myself, I have enough experience to know what I can eat and what I drink.*	Steve
		*It is a personal effort… you learn on your own, from the internet, from reading, from renal patient groups... Mainly I learn from the internet… doctors do not tell you much.*	Marvin
	3b. Professional skills	*I was lucky because I was trained by (states the nurse’s name) and this girl is talented, that is not a compliment, it’s true. She knows very well what one should do and why, and she was very good at conveying knowledge and information. She helped me immensely.*	Adam
		*They did not give me enough information, in order not to scare me… But I think I attained adequate information… I know what I ought to know.*	Belinda
		*What we do not like is that they (nurses) do not inform you when they replace filters, or when one has to undergo an on-line procedure. They do it without asking permission, which annoys me. When a procedure that might affect you is altered, you have the right to be informed. Sometimes, it takes two, three sessions to notice a change… No information was provided.*	Marvin
		*I do not feel I got any education at all… At the beginning, they gave me a leaflet with nutrition guidelines, a very good leaflet with advice. This included basic information, now if I want to ask something, I go ahead and do it. I did not get any formal education or attend seminars in order to understand the process and the supportive practices, so that I know what to do and what to expect.*	Moira
		*Education was sufficient. There are, though, some organizational problems that impede good organization in this context. I was not trained for the hemodialysis process. Something that could be done in two days, it took me eight months… they could not find two days to train me… there were moving the unit to another place but they had to consider my case and pay some attention to me in terms of training.*	Adam
